# Smoking and nasopharyngeal carcinoma mortality: a cohort study of 101,823 adults in Guangzhou, China

**DOI:** 10.1186/s12885-015-1902-9

**Published:** 2015-11-16

**Authors:** Jia-Huang Lin, Chao-Qiang Jiang, Sai-Yin Ho, Wei-Sen Zhang, Zhi-Ming Mai, Lin Xu, Ching-Man Lo, Tai-Hing Lam

**Affiliations:** School of Public Health, University of Hong Kong, No 21 Sassoon Road, Hong Kong, China; Guangzhou Occupational Diseases Prevention and Treatment Center, No 1 Tianqiang Street, Huang Pu Road West, Tianhe District, Guangzhou, 510620 China

**Keywords:** Nasopharyngeal carcinoma, Smoking, Guangzhou Occupational Cohort, Chinese

## Abstract

**Background:**

Nasopharyngeal carcinoma (NPC), also known as Cantonese cancer, is rare worldwide, but has particularly high incidence in North Africa and Southeast Asia, especially in Guangdong, China, such as Guangzhou. Tobacco causes head and neck cancers, but nasopharyngeal carcinoma is not included as causally related to smoking in the 2014 United States Surgeon General’s report. Prospective evidence remains limited. We used Guangzhou Occupational Cohort data to conduct the first and robust prospective study on smoking and NPC mortality in an NPC high-risk region.

**Methods:**

Information on demographic characteristics and smoking status was collected through occupational health examinations in factories and driver examination stations from March 1988 to December 1992. Vital status and causes of deaths were retrieved until the end of 1999. Cox proportional hazard model was used to assess the association of smoking with NPC mortality.

**Results:**

Of 101,823 subjects included for the present analysis, 34 NPC deaths occurred during the average 7.3 years of follow up. The mean age (standard deviation) of the subjects was 41 (5.7) years. Compared with never smokers, the hazard ratio (HR) of NPC mortality was 2.95 (95 % confidence interval 1.01–8.68; *p* = 0.048) for daily smokers and 4.03 (1.29–12.58; *p* = 0.016) for smokers with more than 10 pack-years of cumulative consumption, after adjusting for age, sex, education, drinking status, occupation and cohort status and accounting for smoking-drinking interaction. The risk of NPC mortality increased significantly with cigarettes per day (p for trend = 0.01) and number of pack-years (p for trend = 0.02).

**Conclusions:**

In this first and largest cohort in a high NPC risk region, smoking was associated with higher NPC mortality. The findings have shown statistically significant dose–response trend between smoking amount and smoking cumulative consumption and the risk of NPC mortality, but due to the small event number, further studies with larger sample size are needed to confirm the findings in the present study. Our results support that smoking is one of the risk factors likely to be causally associated with NPC mortality.

**Electronic supplementary material:**

The online version of this article (doi:10.1186/s12885-015-1902-9) contains supplementary material, which is available to authorized users.

## Background

Nasopharyngeal carcinoma (NPC), also known as Cantonese cancer, has a distinctive geographic variation [1–3]. It is rare worldwide, but the incidence is particularly high in North Africa and Southeast Asia, especially in Guangdong, China (for instance, Guangzhou City). The peak age of NPC is also different in high and low risk populations (high-risk: 40–55 years, low-risk: 15–24 and 65–79 years), but the male to female ratio is 2.5–3 to 1 across populations consistently. Tobacco is classified as a group 1 carcinogen by the International Agency for Research on Cancer (IARC). It is a well-known causal factor for head and neck cancers, except NPC.

In the past 3 to 4 decades, 43 case–control studies and 6 cohort studies (including our Guangzhou Occupational Cohort Study) have reported results on the association between tobacco use and NPC. In 2012, IARC reported a causal association of smoking with NPC based on 14 case–control studies and 6 cohort studies [4]. A meta-analysis [5] included 28 case–control and 4 cohort studies found a higher risk of NPC in ever-smokers than never smokers (pooled odds ratio (OR): 1.60, 95 % confidence interval (CI): 1.38–1.87). However, the 2014 United States (US) Surgeon General Report [6] and the 2012 China Tobacco Hazard Report [7] were undecided on whether the association between smoking and NPC is causal.

The quality of these case–control and cohort studies varied. About two-thirds of them were conducted in low or medium risk areas. Much of the evidence came from case–control studies, which might be subject to recall bias with over-estimated odds ratios. Prospective studies reporting significant associations between smoking and NPC risk are scarce.

The Guangzhou Occupational Cohort was among the largest cohorts included in the Asia Pacific Cohort Studies Collaboration with numerous publications [8–10]. The Guangzhou Occupational Cohort has the largest number of NPC deaths in high risk areas, whereas the numbers in the other cohorts were too small for further analysis. The aim of the present paper was to examine whether baseline smoking predicted NPC mortality in Chinese adults in Guangzhou, which is a high NPC risk region.

## Methods

### Subjects

The Guangzhou Occupational Diseases Prevention and Treatment Center established a regular occupational health examination system to monitor the workers’ health and occupational hazards [11]. Depending on their severity of occupational exposure, workers from factories, and drivers renewing their driver’s license were required to undergo biannual, annual or biennial medical examinations to certify their fitness. The examination results were recorded in standardized data collection forms by physicians [12].

The Guangzhou Occupational Cohort was established from the information in the standardized forms. A total of 165,634 subjects (129,135 men and 36,499 women) were included from 399 factories and 11 driver examination stations from 1988 to 1992. These factories and stations covered about 67 and 75 % of all eligible factories and Guangzhou resident drivers, respectively. Hence, the whole cohort comprised of 2 sub-cohorts: workers (*n* = 82,160) and drivers (*n* = 83,474). Details of the methods were reported elsewhere [11, 12] and this cohort has contributed several publications on smoking and related mortality to the Asia Pacific Cohort Studies Collaboration [8–10].

The vital status and cause of death of all subjects were ascertained from factories, Public Health Bureau Statistics Office, funeral homes and local police stations through December, 1998 in the worker cohort and September, 1999 in the driver cohort by two physicians and double checked by a medically qualified epidemiologist [12]. During the follow up period, 95 NPC deaths (76 workers, 19 drivers) had occurred [12]. All coders were blinded to the subjects’ baseline information, and the International Classification of Disease 9^th^ revision (ICD-9) was used to classify the causes of death [11–13].

Ethics approval (for all sites and participants) was obtained from the Ethics Committee, Faculty of Medicine, The University of Hong Kong. Permission to use data was granted by Guangzhou Occupational Diseases Prevention and Treatment Centre.

### Analytical cohort and exposure

The present analysis excluded subjects who had cancers (including NPC), cardiovascular diseases, respiratory diseases and other diseases at baseline; deaths within two years; and those who had no information on daily smoking amount or duration. The final analytical cohort included 101,823 subjects (86,269 men and 15,554 women) (Additional file 1: Figure S1).

Information on demographic characteristics, smoking and drinking status, occupational exposures, and medical history was collected at baseline [11–13]. Data on smoking included smoking status (never smokers, occasional smokers, daily smokers and ex-smokers), number of cigarettes smoked per day, and smoking duration (years of smoking). A daily smoker was defined as one who smoked at least one cigarette per day for at least six months [11]. The numbers of occasional (5 %) and ex-smokers (0.6 %) were too small for statistical analysis and were excluded from the analysis.

Cumulative tobacco consumption (pack-years) was computed as follows: number of pack-years = (number of cigarettes smoked per day × number of years of smoking)/20 (20 cigarettes per pack).

### Statistical methods

Cox proportional hazard model was used in SPSS 20.0 to estimate hazards ratios (HRs) and 95 % confidence intervals (CIs) of NPC mortality for tobacco use adjusting for age, sex, education level, drinking status, occupation and cohort status. P for trend was tested by regressing the smoking variables (e.g., 1 for never smokers, 2 for 1–14 cigarettes/day, and 3 for 15+ cigarettes/day) as continuous variables in the Cox model.

Alcohol is a well-known risk factor of NPC which can modify the effect of smoking on NPC risk. [14] Therefore, interaction between smoking and drinking status was accounted for the analysis. Smoking and alcohol exposures were also stratified based on the paper by Ferreira Antunes et al. [15]. The proportional hazards assumption was checked by visual inspection of plots of log (−log S) against time, where S is the estimated survival function.

## Results

In the final analytical cohort of 101,823 subjects, the total follow up was 746,159 person-years and the mean follow up duration was 7.3 years. The subjects were aged 30 to 87 years with a mean (standard deviation) age of 41.0 (5.7) years. Thirty four NPC deaths were observed (30 male deaths, 4 female deaths).

Table 1 shows that about 48 % men and almost 54 % women were aged 35 to 39 years. About 77 % had secondary education. Almost all (93 %) were rated as healthy or fairly healthy by physicians. Daily smoking rate was 53.3 % in men, but 0.6 % in women. Over 80 % in men and almost 98 % women were never drinkers. Missing data were about 1 % in all variables.

**Table 1:** Characteristics of the 101,823 subjects in the Guangzhou Occupational Cohorts, at baseline (1992)

	Men	Women	Total
	*N* = 86,269 (%)	*N* = 15,554 (%)	*N* = 101,823 (%)
Age, years			
30–34	4568 (5.5)	993 (6.4)	5561 (5.5)
35–39	41,645 (48.3)	8452 (54.3)	50,097 (49.2)
40–44	21,769 (25.2)	4107 (26.4)	25,876 (25.4)
45–49	9663 (25.2)	1488 (9.6)	11,151 (11.0)
50–54	5033 (5.8)	392 (2.5)	5425 (5.3)
55–59	3077 (3.6)	80 (0.5)	3157 (3.1)
60+	514 (0.6)	42 (0.3)	556 (0.6)
Marital status			
Married	85,247 (98.8)	15,143 (97.4)	100,390 (98.6)
Not married	510 (0.6)	345 (2.2)	855 (0.8)
Missing	512 (0.6)	66 (0.4)	578 (0.6)
Education level			
Primary or below	7440 (8.6)	3152 (20.3)	10,592 (10.4)
Secondary	67,405 (78.1)	11,073 (71.2)	78,478 (77.1)
Tertiary	10,894 (12.6)	1222 (7.9)	12,116 (11.9)
Missing	530 (0.6)	107 (0.7)	637 (0.6)
Rank			
Cadre	19,535 (22.6)	3326 (21.4)	22,861 (22.5)
Worker	65,458 (75.9)	12,090 (77.7)	77,548 (76.2)
Missing	1276 (1.5)	138 (0.9)	1402 (1.4)
Health status			
Healthy	71,337 (82.7)	11,666 (75.0)	83,003 (81.5)
Fairly Healthy	9018 (10.5)	2642 (17.0)	11,660 (11.5)
Abnormal	1332 (1.5)	74 (0.5)	1406 (1.4)
Unknown	4346 (5.0)	1061 (6.8)	5407 (5.3)
Missing	236 (0.3)	111 (0.7)	347 (0.3)
Smoking status			
Never smokers	40,119 (46.5)	15,464 (99.4)	55,583 (54.6)
Occasional smokers	114 (0.1)	1 (0.01)	115 (0.1)
Daily smokers	45,988 (53.3)	89 (0.6)	46,077 (45.3)
Ex-smokers	13 (0.02)	0	13 (0.01)
Missing	35 (0.04)	0	35 (0.03)
Drinking status			
Never drinkers	71,340 (82.7)	15,164 (97.5)	86,504 (85.0)
Occasional smokers	6273 (7.3)	341 (2.2)	6614 (6.5)
Daily drinkers	8432 (9.8)	43 (0.3)	8475 (8.3)
Ex-drinkers	27 (0.03)	0	27 (0.03)
Missing	197 (0.2)	6 (0.04)	203 (0.2)

In Table 2, compared with never smokers, the crude HR of NPC mortality from smoking in daily smokers was 3.57 (95 % CI 1.66–7.65; *p* = 0.001), the adjusted HR was 2.95 (1.01–8.68; *p* = 0.048). The crude HR for smoking 15 cigarettes or more per day was 4.52 (2.01–10.14; *p* < 0.001) and the adjusted HR was 4.00 (1.29–12.35; *p* = 0.016). Smoking for 10 years or more showed a crude HR of 3.94 (1.80–8.60; *p* = 0.001) and an adjusted HR of 2.93 (0.97–8.89; *p* = 0.058). The crude and adjusted HR for smoking more than 10 pack-years was 5.52 (2.50–12.20; *p* < 0.001) and 4.03 (1.29–12.58; *p* = 0.016), respectively. All the adjusted HRs above were accounted for smoking-alcohol interaction

**Table 2:** Hazard ratios (HRs) of NPC deaths by smoking status in the combined cohort (both sexes)

	Total person-years of participants	Mortality rate of NPC per 10,000 person-years (95 % CI)	Crude HR (95 % CI)	Model 1 HR (95 % CI)
Never smokers	409,095	0.22 (0.11–0.42)	1.00	1.00
Smoking status				
Daily smokers	337,064	0.74 (0.50–1.10)	3.57 (1.66–7.65)**	2.95 (1.01–8.68)*
Smoking amount (cigarettes/day)				
1–14	162,244	0.49 (0.25–0.99)	2.45 (0.94–6.37)	1.74 (0.45–6.79)
15+	174,820	0.97 (0.60–1.56)	4.52 (2.01–10.14)**	4.00 (1.29–12.35)*
P for trend			<0.001	0.012
Smoking duration (years)				
1–9	87,556	0.46 (0.17–1.22)	2.36 (0.72–7.73)	3.07 (0.69–13.62)
10+	249,508	0.84 (0.55–1.29)	3.94 (1.80–8.60)**	2.93 (0.97–8.89)
P for trend			0.001	0.064
Smoking cumulative consumption (pack-years)				
< 10	181,243	0.33 (0.15–0.74)	1.65 (0.59–4.66)	1.76 (0.45–6.89)
10+	155,821	1.22 (0.78–1.91)	5.52 (2.50–12.20)**	4.03 (1.29–12.58)*
P for trend			<0.001	0.014

Model 1- adjusted by age, sex, education, drinking status, cohort status & occupation (including cadre level workers, general workers, and drivers), accounted for smoking-drinking interaction

*p < 0.05, **p < 0.01

Significant trends suggesting increased risk with daily smoking amount (p for trend = 0.01) and cumulative consumption (pack-years) (p for trend = 0.01) were observed in the adjusted HRs of Model 1, but the trend was of borderline significance for smoking duration (p for trend = 0.06) (Table 2, Model 1). Note that the HRs in the lower exposure group did not significantly different from unity.

The joint effects of smoking and drinking observed for NPC mortality risk were assessed in Table 3. The use of the interaction term resulted in an increase in the values of the HRs of almost all the smoking-related variables. However, the number of NPC deaths was small (*n* = 27) and the 95 % CI were wide.

**Table 3:** Individual and joint effects of smoking and drinking on NPC adjusted for age, sex, education, cohort & occupational status

Category	Total person-years of participants	Mortality rate of NPC per 10,000 person-years (95 % CI)	No of deaths	Model 2 HR (95 % CI)
Smoking and drinking status				
Never smoker and never drinker	390,148	0.21 (0.10–0.41)	8	1.00
Never smoker and daily drinker	6265	1.60 (0.22–11.33)	1	4.19 (0.47–37.22)
Daily smoker and never drinker	240,998	0.50 (0.28–0.88)	12	2.95 (1.01–8.68)*
Daily smoker and daily drinker	56,597	1.06 (0.48–2.36)	6	3.38 (0.95–11.97)
Smoking amount and drinking status (cigarettes/day)^a^				
Never smoker and never drinker	390,148	0.21 (0.10–0.41)	8	1.00
Never smoker and daily drinker	6265	1.60 (0.22–11.33)	1	4.49 (0.51–39.90)
Level 1 smoking amount and never drinker	125,001	0.32 (0.12–0.85)	4	2.15 (0.56–8.18)
Level 1 smoking amount and daily drinker	21,113	/	0	/
Level 2 smoking amount and never drinker	115,997	0.53 (0.28–1.02)	8	3.55 (1.12–11.31)*
Level 2 smoking amount and daily drinker	35,485	1.28 (0.58–2.86)	6	4.86 (1.34–17.61)*
Smoking duration and drinking status (years)^b^				
Never smoker and never drinker	390,148	0.21 (0.10–0.41)	8	1.00
Never smoker and daily drinker	6265	1.60 (0.22–11.33)	1	4.21 (0.47–37.41)
Level 1 smoking duration and never drinker	71,958	0.42 (0.13–1.29)	3	3.52 (0.81–15.34)
Level 1 smoking duration and daily drinker	9877	/	0	/
Level 2 smoking duration and never drinker	169,040	0.53 (0.28–1.02)	9	2.67 (0.87–8.23)
Level 2 smoking duration and daily drinker	46,720	1.28 (0.58–2.86)	6	3.61 (1.01–12.93)*
Smoking cumulative consumption and drinking status (pack-years)^c^				
Never smoker and never drinker	390,148	0.21 (0.10–0.41)	8	1.00
Never smoker and daily drinker	6265	1.60 (0.22–11.33)	1	4.65 (0.52–41.41)
Level 1 smoking cumulative consumption and never drinker	143,711	0.28 (0.10–0.74)	4	2.10 (0.55–8.11)
Level 1 smoking cumulative consumption and daily drinker	22,306	/	0	/
Level 2 smoking cumulative consumption and never drinker	97,286	0.82 (0.41–1.64)	8	3.69 (1.15–11.82)*
Level 2 smoking cumulative consumption and daily drinker	34,291	1.75 (0.79–3.89)	6	4.77 (1.30–17.50)*

Model 2 – adjusted for age, sex, education, cohort & occupational status with smoking and alcohol interaction terms

*p < 0.05

^a^Level 1: smoked 1–14 cigarettes per day, Level 2: smoked more than 15 cigarettes per day

^b^Level 1: 1–9 years of smoking duration, Level 2: more than 10 years of smoking duration

^c^Level 1: less than 10 pack-years of smoking cumulative consumption, Level 2: more than 10 pack-years of smoking cumulative consumption

The differences between the HRs in the two sub-cohorts were tested by including the interaction term of cohort status by smoking. The *p* value for the interaction term was 0.44, indicating that the association of smoking and NPC mortality did not vary by sub-cohort status.

## Discussion

We showed that higher daily smoking amount and greater cumulative consumption being associated with higher risk of NPC mortality. An earlier paper from the Guangzhou Occupational Study showed some preliminary results of the association in 2004 [13]. However, this previous analysis only focused on the worker sub-cohort (*n* = 82,160), compared the risk between ever and never smokers and did not report dose–response results. The present study included the driver sub-cohort. We carefully used the most appropriate exclusion criteria for analysis and provided more robust and the first prospective evidence with dose–response relation from the largest cohort in an NPC endemic area.

According to the World Health Organization (WHO) [16], the 3 histological types of NPC have different distribution in high and low risk areas. Over 90 % of NPC cases in high-risk areas are undifferentiated carcinoma (Type III), while squamous-cell carcinoma (Type I) is the major histologic type in low-risk areas [5, 17]. Previous studies, including a meta-analysis [5], demonstrated that the association between smoking and NPC was stronger in squamous-cell carcinoma (Type I, main cell type in low-risk population) than that in undifferentiated carcinoma (Type III, main cell type in high-risk population) [5, 18]. A slightly higher risk of squamous-cell NPC (pooled OR 2.20, 95 % CI 1.63–2.98) than that of undifferentiated NPC (pooled OR 1.27, 95 % CI 0.98–1.66) was found in Xue’s meta-analysis from 4 case–control studies, which might be subject to recall bias resulting in higher ORs. Studies included in the meta-analysis also had the heterogeneity problem (*p* < 0.01, I^2^ = 86). Unfortunately, we did not have histology data for the NPC deaths. But other studies in Guangzhou and other high-risk areas have shown that the predominant type of NPC is undifferentiated carcinoma (which accounts for 90 % of cases in endemic regions) [3, 19, 20]. Our results may indicate that smoking has a stronger (than previously expected) association with NPC mortality in high-risk areas as well as those in low-risk areas.

Among all 6 cohort studies [13, 17, 18, 21–23] on smoking and NPC, Guangzhou had the highest age standardized incidence rate (ASIR) of NPC in men (22.2/100,000) and in women (9.9/100,000) [24], which was twice as high as those in intermediate NPC risk regions of Singapore (10.5/100,000 in men, 5.4/100,000 in women) [17] and Taiwan (9.02/100,000 in men, 2.79 in women) [25] and more than 20 times higher than those in low risk regions of the US and United Kingdom (1/100,000).

As mentioned above, only six cohort studies including the present Guangzhou cohort [13] had reported results on NPC and tobacco use [17, 18, 21–23]. Among the two cohort studies conducted in low-risk areas, only the one from the US [18] reported a significant trend with daily smoking amount, but this study did not find a significant trend for smoking duration and did not report results on cumulative consumption. The other study from the British doctors cohort [23] did not report any significant results because of the small number of NPC (*n* = 4). In endemic areas, two cohort studies in Taiwan [21, 22] and one cohort study in Singapore [17] reported a significant increase in NPC risk in heavy and chronic smokers, but only the Singapore study showed a dose–response relation by smoking duration, whereas the two Taiwan studies did not show any significant trends.

Our results have provided new prospective evidence that heavy and chronic smokers had significantly increased risk of NPC mortality, which are consistent with most case–control studies and the 4 cohort studies from Taiwan, Singapore and the US [5, 17, 18, 22]. The adjusted HR from our study was 3.26 (1.14–9.36) in heavy smokers who smoked more than 15 cigarettes per day, which was consistent (with overlapping 95 % CI) with that of 3.3 (0.8–14.0) from Liaw et al. in Taiwan. We observed significant trends suggesting a dose–response relation for daily smoking amount, which was consistent with the findings from the US cohort study. [18] Moreover, our study was the first to observe a significant trend suggesting dose–response relation for cumulative tobacco use among all cohort studies above. But the limited number of NPC deaths restricted our grouping of exposure levels (only three exposure levels). The significant trends might be due to the large HRs in the group with the greatest exposure. Because the HRs in the low exposure group did not significantly different from unity, a threshold level below which smoking is not associated with increased risk is possible and cannot be ruled out from our study. Future studies with larger sample size are needed to confirm our findings.

Compared with other cohort studies, our study used lower smoking level to define heavy and chronic smokers (more than 10 pack-years, more than 10 years smoking or 15 cigarettes per day), but our results still showed a stronger and significant association between smoking and NPC mortality. Despite the different definitions of heavy exposure, our results are consistent with those of the meta-analysis by Xue et al. [5].

We recognize the limitations of the small number of NPC deaths (especially in women) and the wide 95 % confidence intervals of the risk estimates, the lack of data on nonfatal new cases and the short follow-up. However, there was only one cohort study [18] which had more NPC deaths (*n* = 48) than ours. We are now planning for a further follow-up of the cohort.

Because alcohol consumption and smoking are important NPC risk factors [14], alcohol status and smoking-drinking interaction were accounted for in the present analysis. The *p* value for the smoking-drinking interaction was 0.26, which was not significant. The use of smoking and alcohol interaction term resulted in an increased in HRs for combined exposure of alcohol intake and smoking HRs, which suggested an interaction between smoking and drinking, but the 95 % CIs were very wide. Due to the small event number, we cannot fully investigate the joint effects of smoking and drinking and the risk of NPC. Further investigations with more NPC deaths are needed. Although we suspect some drivers did not disclose their drinking habit or under-reported their consumption during the driver license renewal, the results were similar after including alcohol data.

Sensitivity analysis without female subjects and alcohol adjustment were performed in Additional file 1: Table S1. The results of the sensitivity analyses were similar to the main results above.

In the present analysis, we excluded subjects with NPC or other diseases at baseline to reduce bias from reverse causation (people with NPC or diseases would be more likely to quit smoking). These strict exclusion criteria probably allowed the better detection of the association with smoking in the analysis.

Despite its declining incidence and mortality in the past decades [2], NPC is still among the top 10 common cancers in epidemic areas, such as Guangzhou [26] and Hong Kong [27]. The present study should provide convincing new evidence to support causal inference on the association of smoking with NPC mortality.

China is still in the second stage of tobacco epidemic [28], and tobacco control is a major public health concern. Guangdong not only has the most high-risk population of NPC, but also has a large number of smokers in China (about 16 million, based on the estimated smoking prevalence by Su et al.) [29]. Due to the time gap between smoking and disease occurrence, the consequences of tobacco use in smokers will continue to expand, including NPC if the association is causal. Tobacco control in high NPC risk areas, including smoking cessation, should have additional benefits in reducing NPC.

Further follow-up of the present cohort, including fatal and nonfatal NPC is warranted. There are many cohort studies in China mainland in the Asia Pacific Cohort Studies Collaboration and elsewhere which can be used for further follow-up and analysis. Detailed analyses, including meta-analyses based on pooled individual data are low cost studies to examine smoking and other risk factors of NPC. Information on the histological types of NPC, which is often lacking in most cancer registries and NPC epidemiological studies, is essential to estimate the histology-specific risk of NPC for smoking, and its interaction with other risk factors (such as EB virus) and to study the mechanisms of carcinogenesis.

## Conclusions

In our study, heavier and chronic smokers had significantly higher risk of NPC mortality. Significant trends suggesting dose–response relationships of smoking amount and smoking cumulative consumption and the risk of NPC mortality were observed in our study, but more prospective cohorts with more NPC deaths are required to confirm our results, or to examine whether there is a threshold level of exposure, which has important implications for cancer prevention. The results from this first and largest prospective study in a high NPC risk region support that smoking is one of the risk factors likely to be causally associated with NPC mortality. Strong tobacco control measures are needed to motivate smokers to stop smoking.

## Additional file

Additional file 1: Table S1. Hazard ratios (HRs) of NPC deaths by smoking status in the combined cohort (men). **Figure S1**: The flow chart of the analytical cohort selection in Guangzhou Occupational Cohort (GZOC). (DOCX 29 kb)

## Abbreviations

NPC: Nasopharyngeal carcinoma
HR: Hazard ratio
IARC: International Agency for Research on Cancer
OR: Odds ratio
CI: Confidence interval
US: United States
WHO: World Health Organization
ASIR: Age standardized incidence rate
