# Supplementary material for: Smoking and nasopharyngeal carcinoma mortality: a cohort study of 101,823 adults in Guangzhou, China
Source: BMC Cancer. 2015 Nov 16;15:906. doi: 10.1186/s12885-015-1902-9 (PMC4647498; doi:10.1186/s12885-015-1902-9)
Supplement: Additional file 1: Table S1. — Hazard ratios (HRs) of NPC deaths by smoking status in the combined cohort (men). Figure S1: The flow chart of the analytical cohort selection in Guangzhou Occupational Cohort (GZOC). (DOCX 29 kb) [file 12885_2015_1902_MOESM1_ESM.docx]

**Additional file 1:**

| **Table S1: Hazard ratios (HRs) of NPC deaths^#^ by smoking status in the combined cohort (men)** | | | | |
| --- | --- | --- | --- | --- |
|  | **Total person-years of participants** | **Mortality rate of NPC per 10,000 person-years (95% CI)** | **Crude HR (95% CI)** | **Adjusted HR^ (95%CI)** |
| **Never smokers** | 290281 | 0.21 (0.09-0.46) | 1.00 | 1.00 |
| **Smoking status** |  |  |  |  |
| Daily smokers | 335988 | 0.70 (0.48-1.07) | 3.31 (1.35-8.11)** | 2.70 (1.09-6.67)* |
| **Smoking amount (cigarettes/day)** | |  |  |  |
| 1-14 | 161643 | 0.43 (0.21-0.91) | 2.11 (0.71-6.27) | 2.16 (0.72-6.46) |
| 15+ | 174345 | 0.98 (0.61-1.57) | 4.36 (1.71-11.10)** | 3.04 (1.18-7.87)* |
| P for trend |  |  | 0.001 | 0.02 |
| **Smoking duration (years)** | |  |  |  |
| 1-9 | 87351 | 0.45 (0.17-1.22) | 2.35 (0.66-8.33) | 3.56 (0.97-13.02) |
| 10+ | 248637 | 0.80 (0.52-1.25) | 3.62 (1.45-9.04)** | 2.55 (1.00-6.46)* |
| P for trend |  |  | 0.005 | 0.06 |
| **Smoking cumulative consumption (pack-years)** | |  |  |  |
| <10 | 180750 | 0.33 (0.15-0.74) | 1.65 (0.53-5.10) | 2.05 (0.65-6.47) |
| 10+ | 155238 | 1.16 (0.73-1.84) | 5.08 (2.01-12.88)** | 3.07 (1.18-7.96)* |
| P for trend |  |  | <0.001 | 0.02 |
| ^#^2 NPC cases with missing data of smoking duration were excluded | | | | |
| ^adjusted by age, education, cohort status & occupation (including cadre level workers, general workers, and drivers ) | | | | |
| * p<0.05, **p<0.01 | | | | |

**Figure S1: The flow chart of the analytical cohort selection in Guangzhou Occupational Cohort (GZOC)**

Excluded ex-smokers (0.6%) and occasional smokers (5%)

Excluded subjects had cancers, cardiovascular diseases, respiratory diseases and other diseases at baseline; deaths within two years; and those who had no information on smoking

Number in analytical cohort:

Men: 86268 (30 NPC deaths)

Women: 15554 (4 NPC deaths)

Total: 101823 (34 NPC deaths)

Number of men in GZOC:

Men: 119287 (74 NPC deaths)

Women: 34302 (19 NPC deaths)

Total: 153589 (93 NPC deaths)

Number of subjects in Guangzhou Occupational Cohort

Men: 129135 (76 NPC deaths)

Women: 34499 (19 NPC deaths)

Total: 165634 (95 NPC deaths)
